# Reviewed article: Silva MT, Amado DM, Rocha PRS, Barreto JOM. Integrative and Complementary Health Practices for Chronic Pain: A Summary of Clinical Guideline Recommendations. Epidemiol Serv Saude. 2025:34;e20240771.

**DOI:** 10.1590/S2237-96222025v34e20240771.a

**Published:** 2025-08-11

**Authors:** Pedro Henrique Brito da Silva

**Affiliations:** Secretaria Municipal de Saúde de Senador Canedo

**Table:** 

Rating	Excellent	Good	Average	Below average	Poor
Interest		✓			
Quality				✓	
Originality				✓	
Overall				✓	

**Table:** 

Questionnaire	Yes	No	Not applicable
Does the manuscript contain new and significant information to justify publication?	✓		
Does the Abstract (Summary) clearly and accurately describe the content of the article?		✓	
Is the problem significant and concisely stated?		✓	
Are the methods described comprehensively?		✓	
Are the interpretations and conclusions justified by the results?		✓	
Is adequate reference made to other work in the field?	✓		

## Manuscript Structure

Length of article is: Adequate

Number of tables is: Adequate

Number of figures is: Adequate

Please state any conflict(s) of interest that you have in relation to the review of this paper (state “none” if this is not applicable): None

## Comments

Caros autores, seguem as minhas sugestões para melhora da escrita do artigo. O tema é relevante, mas, para publicação, são necessárias várias modificações.

Resumo

No resumo, não se utiliza siglas e/ou acrônimos. Todas as palavras devem ser redigidas por extenso nessa seção do artigo científico. A seção dos resultados no resumo está muito pobre e não abarca o objetivo do estudo. Os autores apontam os níveis de evidência das recomendações encontradas. Mas, não torna o resumo atrativo para que os pesquisadores ou qualquer outra pessoa leia o artigo na íntegra. Os autores destacam 8 diretrizes de alta qualidade metodológica. Para a literatura isso é muito importante, haja vista os recorrentes ataques contra a implementação das Práticas Integrativas e Complementares no Sistema Único de Saúde. Sugiro que os autores façam um compilado dessas 8 diretrizes nos resultados do resumo.

O objetivo também está um pouco confuso. Os autores declaram o objetivo: “avaliar as recomendações de diretrizes clínicas de alta qualidade metodológica, para o uso de Práticas Integrativas e Complementares em Saúde (PICS) no manejo da dor crônica em adultos”. Então, os autores afirmam que irão avaliar somente as recomendações de alta qualidade. Porém, os resultados apontados pelos autores incluem também as diretrizes de baixa qualidade metodológica. Sugiro que os autores revisitem o objetivo do estudo, pois prometem fazer uma coisa e estão fazendo outra. Dessa forma, a conclusão do estudo também enfatiza muito mais as recomendações de baixa do que aquelas com alta qualidade metodológica. Mas, o objetivo não é valorar as recomendações de alta qualidade? Ou se quer encontrar, avaliar e analisar todas as recomendações publicadas nas bases de dados científicos?

Com relação ao método, a descrição é objetiva, mas a integração dos detalhes sobre o sistema GRADE e a ferramenta AGREE II poderia ser mais fluida. Sugiro revisar a estrutura para evitar confusão entre as etapas metodológicas. A expressão “revisão rápida” é vaga. Seria útil especificar se há justificativa para esse tipo de revisão e como isso impactou a abrangência da busca. Então, especificar o racional para a escolha de uma “revisão rápida” e detalhar como as diretrizes foram selecionadas e avaliadas. Também é importante clarificar como o sistema GRADE foi usado para definir a qualidade da evidência e a força das recomendações.

Nos resultados, a quantidade de diretrizes incluídas e os critérios de alta qualidade metodológica são informados adequadamente, mas a explicação sobre as “recomendações fracas” precisa de maior clareza. Poderiam explicitar como a força das recomendações foi avaliada. Dessa forma, os pesquisadores devem explicitar as diferenças entre recomendações fortes e fracas, alinhando isso aos critérios metodológicos. Além disso, recomendo justificar a afirmação sobre custo-efetividade, incluindo menção a evidências econômicas ou removendo a afirmação, se não for sustentada pelos dados.

Na conclusão, os autores devem reconhecer explicitamente as limitações do estudo, reforçando que a qualidade limitada da evidência impede conclusões definitivas. Sugiro que os autores apontem a necessidade de mais estudos robustos, destacando o potencial das PICS como uma estratégia complementar promissora. Por fim, não está claro se as diretrizes incluídas são representativas globalmente ou se possuem relevância específica para o contexto do SUS. Isso deveria ser abordado para contextualizar melhor as conclusões. De modo geral, são necessários vários ajustes por parte dos pesquisadores para que esta seção do artigo seja mais coerente e atraente para o leitor.

Introdução

A introdução é bastante problemática e genérica. Os autores redigem várias frases de efeito e sem conexão com os parágrafos. Os autores não explicitam vários pontos necessários para se ter uma boa redação nessa seção do estudo. A relevância do estudo não é destacada ao contexto brasileiro. Por que sabermos mais dessas recomendações seria relevante para a política pública das Práticas Integrativas e Complementares? Sinto falta de uma contextualização das PICS no Brasil, além de uma breve definição de PICS e dor crônica. Citar que em 2017 e 2018, a Política Nacional de Práticas Integrativas foi atualizada. Com isso, as pesquisas envolvendo PICS ganham ainda mais relevância.

Desse modo, os autores também não explicitam a lacuna de pesquisa que o estudo pretende preencher. Os autores trazem também inúmeras vantagens da implementação das PICS. Porém, em nenhum momento foi citado os questionamentos e críticas apontados por pesquisadores sobre algumas pseudociências implementadas e contempladas na política nacional de Práticas Integrativas (PNPIC). Isso seria uma boa justificativa para a execução desse estudo. Mostrar que existem recomendações com alto teor metodológico ajudaria os pesquisadores das PICS a embasar e prosseguir com suas pesquisas e os trabalhadores que as implementam no SUS.

Além disso, os autores não detalham por que uma análise baseada no sistema GRADE sobre diretrizes de PICS para dor crônica é inédita ou necessária. Os autores também não redigem a pergunta de investigação. Toda pesquisa deve deixar clara o que se quer responder com os seus achados. Também seria importante trazer a hipótese, a resposta provisória a essa pergunta. O que os autores esperam encontrar na literatura? Esperam recomendações fortes ou fracas? Dessa forma, a redação da discussão pode se tornar mais interessante, pois será que os autores encontraram resultados diferentes daquilo que acreditavam incialmente? Os autores também elencam vários objetivos. Eles estão bastantes desconexos com a introdução. Sugiro a redação de um objetivo geral mesmo. Em resumo, os autores poderiam fortalecer a introdução explicitando melhor a lacuna de pesquisa, formulando uma pergunta de investigação clara e conectando-a com os objetivos do estudo. Além disso, é importante melhorar a organização do texto para evitar repetição de ideias e assegurar uma linha de raciocínio coesa.

Métodos

A justificativa do uso de uma revisão rápida está apresentada, mas falta especificidade sobre como os “atalhos metodológicos” foram implementados sem comprometer a qualidade dos resultados. Isso é particularmente importante porque revisões rápidas podem ser criticadas por limitações metodológicas. Os autores poderiam explicar melhor quais compromissos foram feitos (como restrição de bases de dados ou simplificação na triagem) e justificar essas escolhas.

As revisões de escopo rápidas, os métodos podem utilizar a sigla PCC, em que “P” representa a população em estudo, “C” contempla os termos de conceito relacionados ao fenômeno da pesquisa e “C” se refere aos termos relacionados ao contexto. Sugiro que os autores avaliem a possibilidade de se incluir o PCC para embasar a construção da pergunta de investigação e para criação do mapeamento dos conceitos. Dessa forma, conseguimos justificar a estratégia de busca adotada para se chegar aos resultados da pesquisa.

Os critérios de elegibilidade estão bem definidos, mas a escolha de incluir diretrizes de qualquer idioma e data de publicação requer maior explicação. A ausência de restrições de idioma pode ser positiva, mas os autores deveriam descrever se houve tradutores envolvidos para garantir a compreensão adequada das diretrizes em outros idiomas. Além disso, aceitar qualquer data de publicação pode ser problemático, pois diretrizes mais antigas podem conter evidências desatualizadas. Uma justificativa para essa inclusão mais ampla seria relevante.

A seleção das fontes de informação (Medline, Embase e Scopus) é apropriada, considerando que são bases amplamente reconhecidas para revisão de literatura em saúde. Contudo, a exclusão de bases como Cochrane Library ou LILACS, que também indexam materiais relevantes, deveria ser discutida. A falta de bases regionais pode limitar a inclusão de diretrizes adaptadas a contextos locais, como o SUS.

A estratégia de busca é mencionada, mas a descrição deixa margem para críticas. Não há informações sobre se a estratégia de busca foi desenvolvida com apoio de um bibliotecário ou especialista em revisão sistemática, o que é uma recomendação do PRISMA. Além disso, não é mencionado se houve testes prévios da estratégia para avaliar sua sensibilidade e especificidade. A Tabela 1, referida no texto, deveria ser mencionada com mais ênfase, detalhando os termos usados, operadores booleanos e ajustes específicos para cada base.

O processo de seleção segue boas práticas ao adotar a triagem em duas etapas por revisores independentes, com resolução de divergências por consenso. No entanto, seria relevante esclarecer se foi utilizado um software de revisão (como Covidence ou Rayyan) para gerenciar os registros e se os revisores receberam treinamento padronizado para aplicar os critérios de elegibilidade.

O processo de coleta de dados está descrito com clareza, mas a confiabilidade do método de extração poderia ser fortalecida com menção a testes-piloto da planilha de coleta e índices de concordância entre os revisores. A ausência de informações sobre como conflitos de extração foram resolvidos é uma lacuna.

A descrição da avaliação do risco de viés das diretrizes clínicas com o AGREE II é detalhada e bem fundamentada, mas seria importante mencionar como os critérios foram ponderados na análise final. Por exemplo, diretrizes que atendem a maior parte dos critérios metodológicos recebem maior peso nas conclusões? Além disso, seria útil descrever se os revisores foram treinados para aplicar a ferramenta e se houve alguma verificação da consistência entre eles.

A avaliação da certeza das evidências, com base na qualidade metodológica das diretrizes e nos critérios do sistema GRADE, é adequada. Contudo, a ausência de explicação detalhada sobre como a análise qualitativa foi realizada enfraquece essa etapa. Por exemplo, que critérios objetivos foram usados para interpretar a combinação das recomendações? Foram utilizadas categorias ou descritores previamente definidos?

No geral, os métodos são bem descritos e mostram aderência a boas práticas, mas algumas melhorias são necessárias para aumentar a transparência, robustez e alinhamento com o checklist PRISMA. Sugere-se que os autores:

1. Justifiquem as escolhas metodológicas específicas da revisão rápida.

2. Expandam a explicação sobre a estratégia de busca e inclusão de bases adicionais.

3. Detalhem melhor os métodos de extração de dados e avaliação do risco de viés.

4. Esclareçam os critérios para análise qualitativa das recomendações e os procedimentos usados para combinar os resultados de diferentes diretrizes.

Resultados

O processo de seleção é adequadamente detalhado, com a inclusão de uma Figura PRISMA para ilustrar as etapas. No entanto, seria relevante discutir se houve problemas comuns durante a triagem, como falta de clareza nos critérios de elegibilidade ou dificuldades em obter textos completos. Além disso, a inclusão de 19 artigos para 18 diretrizes sugere que alguns artigos podem descrever a mesma diretriz; esse detalhe poderia ser melhor explicado para evitar confusão.

A descrição das características das diretrizes incluídas (Tabela 2) é abrangente, destacando a diversidade de condições e intervenções abordadas. No entanto, seria útil incluir uma análise mais detalhada sobre a distribuição geográfica e contextual das diretrizes. Por exemplo, há diretrizes adaptadas especificamente para sistemas públicos de saúde, como o SUS, ou focadas em populações vulneráveis? Isso ajudaria a entender a aplicabilidade no contexto brasileiro.

A avaliação da qualidade metodológica das diretrizes (Tabela 3) é uma contribuição importante, especialmente por identificar lacunas em aspectos críticos, como métodos de busca e relação entre evidências e recomendações. Entretanto, a análise poderia ir além das categorias “alta”, “moderada” e “baixa”, detalhando quais critérios específicos do AGREE II foram mais frequentemente problemáticos. Isso permitiria identificar padrões que poderiam orientar melhorias no desenvolvimento de diretrizes futuras.

A síntese das recomendações (Tabela 4) é clara e informativa, especialmente ao diferenciar a qualidade da evidência e a força das recomendações para diferentes modalidades de PICS e condições de dor crônica. No entanto, a predominância de evidências de baixa a moderada qualidade e recomendações fracas deveria ser explorada em maior profundidade. Por exemplo, os autores poderiam discutir os motivos mais comuns para a baixa qualidade das evidências, como heterogeneidade nos estudos primários ou falta de ensaios clínicos robustos.

Embora acupuntura, osteopatia, quiropraxia, yoga e tai chi sejam destacados como intervenções promissoras, os autores poderiam expandir a análise para discutir por que essas modalidades apresentam maior força de recomendação em algumas diretrizes. Há maior volume de estudos para essas intervenções? Elas têm mecanismos de ação mais bem compreendidos? Esse tipo de análise agregaria valor aos resultados.

A discussão sobre a aplicabilidade das recomendações é limitada. Os autores mencionam que as diretrizes enfatizam o papel das preferências do paciente, mas não abordam como isso poderia ser operacionalizado em sistemas públicos como o SUS. Por exemplo, como as PICS poderiam ser integradas a serviços já existentes, ou quais barreiras e facilitadores podem ser esperados?

Por fim, a análise sobre a variabilidade na confiança nas diretrizes poderia ser mais desenvolvida. Embora algumas diretrizes tenham maior grau de confiança, a força das recomendações ainda é majoritariamente fraca. Isso levanta questões importantes sobre o equilíbrio entre rigor metodológico e aplicabilidade prática, que poderiam ser exploradas nos resultados ou na discussão subsequente.

De modo geral, os quadros podem ser melhor apresentados. Sugiro a conversação de tabelas para os quadros, inicialmente. As tabelas são usadas quando se tem valores numéricos. Então, os quadros se adequam mais para o tipo de dados da pesquisa. Elas podem ser mais atraentes ao leitor e ser de mais fácil compreensão. Os quadros podem ser apresentados com mais linhas verticais e horizontais, dispostas de modo que facilitem a visualização dos dados pelo leitor. A maneira como os autores organizaram dificulta a leitura desses importantes elementos para a compreensão do manuscrito.

Discussão

Discussão bastante pobre. Essa seção deveria ser iniciada com a retomada do objetivo. Além disso, é interessante dizer se a resposta provisória a pergunta de investigação foi contemplada ou não. Dizer o porquê de se ter acontecido a confirmação da resposta esperada ou não. A discussão destaca adequadamente as limitações da síntese de evidências, incluindo a heterogeneidade das diretrizes e a predominância de evidências de qualidade moderada ou baixa. Essas limitações são pertinentes e reforçam a necessidade de pesquisas mais robustas para fortalecer as conclusões e a formulação de recomendações mais consistentes. No entanto, a discussão poderia explorar de forma mais aprofundada os impactos potenciais de vieses de publicação e a possível exclusão de diretrizes relevantes não indexadas nas bases utilizadas, considerando que esses fatores podem influenciar significativamente a representatividade das conclusões.

A análise do papel das PICS no manejo da dor crônica é enriquecida pela menção ao custo-efetividade, segurança e perfil multidimensional dessas práticas, incluindo o potencial para melhorar a funcionalidade física e emocional, qualidade de vida e bem-estar psicológico. A inclusão de exemplos concretos, como a integração da acupuntura na Política Nacional de Práticas Integrativas e Complementares no SUS, confere um caráter aplicável às conclusões, especialmente no contexto brasileiro. Entretanto, a argumentação poderia ser fortalecida com dados mais detalhados sobre a efetividade dessas práticas específicas no SUS e sua relação com os custos e a satisfação dos pacientes.

A discussão também acerta ao enfatizar a necessidade de capacitação de profissionais do SUS em práticas como yoga e meditação, abordagens que têm potencial para serem oferecidas de maneira descentralizada em diferentes níveis de atenção. Contudo, faltou um maior aprofundamento sobre os desafios práticos e logísticos para implementar essa capacitação e expandir o acesso às PICS no sistema público de saúde. Além disso, a relação entre as PICS e o fortalecimento da autonomia do paciente poderia ser melhor explorada, com base em evidências existentes ou lacunas que exigem investigação futura.

A menção ao protocolo de manejo da dor crônica da Secretaria Municipal da Saúde de São Paulo é um ponto relevante, pois demonstra a integração prática das PICS em contextos específicos. Entretanto, seria imperioso expandir a análise para discutir como essa experiência pode ser replicada ou adaptada em outras regiões do país, considerando as diferenças de infraestrutura e recursos no SUS. Além disso, a discussão poderia considerar uma análise crítica mais detalhada dos desfechos relatados nesse protocolo e sua correspondência com as diretrizes internacionais incluídas na revisão.

Por fim, a conclusão sintetiza bem os principais achados, reafirmando que, embora as PICS sejam promissoras, há necessidade de evidências mais sólidas para embasar recomendações robustas. O texto destaca o papel dos profissionais de saúde na integração dessas práticas com a experiência clínica e as preferências dos pacientes, o que é essencial para um cuidado centrado na pessoa. A sugestão de ampliar o acesso às PICS no SUS e investir em pesquisas brasileiras é pertinente, mas a argumentação poderia ser fortalecida com exemplos concretos de iniciativas de pesquisa ou políticas públicas em andamento, que poderiam servir como modelos ou oportunidades de avanço.

Visão geral e decisão

O artigo possui um tema muito relevante para o setor saúde brasileiro. Os procedimentos oferecidos pelo SUS devem ser embasados cientificamente. Essas recomendações são importantes para as PICS e devem ser mais notabilizadas aos profissionais de saúde. Essa revisão rápida pode ser usada para fundamentar pesquisas e discussões acerca das PICS ofertadas pelo sistema público de saúde.

Porém, o artigo ainda está imaturo. O manuscrito carece de uma ampla revisão. A inteligência artificial pode ajudar nisso. Mas, ela não pode fazer pelos autores. Sinto falta da escrita “humana”. A inteligência artificial poderia ser usada somente para correção de ortografia e melhora da escrita. Ao iniciar a leitura, já senti que a IA foi usada. A escrita usada pelos autores é robotizada, o que identifica a presença da IA. Então, o uso da IA é permitido pela revista. Mas, ela pode ser usada de um modo mais sutil e perspicaz. Além disso, os resultados e discussão ainda precisam ser fortificadas. O periódico científico é de grande circulação e merece que o texto dessas seções seja reescrito, melhorado. Precisam ser mais claros.

Diante disso, sugiro a uma revisão maior.

